# Intestinal Protozoa in Domestic Cats (Carnivora: Felidae, *Felis catus*) in Northwestern Iran: A Cross-Sectional Study with Prevalent of Microsporidian and Coccidian Parasites

**Published:** 2019

**Authors:** Mehdi MOHEBALI, Zabiholah ZAREI, Khadijeh KHANALIHA, Eshrat Beigom KIA, Afsaneh MOTAVALLI-HAGHI, Jaber DAVOODI, Fathemeh TARIGHI, Mahya KHODABAKHSH, Mostafa REZAEIAN

**Affiliations:** 1. Department of Medical Parasitology and Mycology, School of Public Health, Tehran University of Medical Sciences, Tehran, Iran; 2. Research Center of Zoonoses, Tehran University of Medical Sciences, Tehran, Iran; 3. Research Center of Pediatric Infectious Diseases, Institute of Immunology and Infectious Diseases, Iran University of Medical Sciences, Tehran, Iran; 4. Department of Veterinary Parasitology, Zanjan Branch, Islamic Azad University, Zanjan, Iran; 5. Department of Internal Diseases, Faculty of Veterinary Medicine, University of Tehran, Tehran, Iran; 6. Center for Research of Endemic Parasites of Iran (CREPI), Tehran University of Medical Sciences, Tehran, Iran

**Keywords:** Intestinal protozoa, Cats, Iran

## Abstract

**Background:**

In this study, some microsporidial and coccidian parasites were isolated from 103 domestic cats in the Meshkin Shahr area, northwestern Iran during the Jun 2014 to Jun 2015, and their genera were identified using parasitological methods with emphasis on their zoonotic importance.

**Methods:**

One hundred and three fecal samples of domestic cats were collected and preserved in formalin (10%) and conserved in phosphate buffer saline solution, finally examined by microscopy after formalin-ether concentration and specific staining. Preservation in dichromate potassium (2.5%) was performed for all coccidian positive samples and then sporulated coccidian oocysts were investigated.

**Results:**

The detected parasites were *Isospora* spp. 6/103(5.8%). Microsporidian spores were identified in 46/103 (44.6%) of all samples post-stained by the aniline blue staining method.

**Conclusion:**

Microsporidial infections were more prevalent in domestic cats. Further studies are needed in the identification of microsporidial spores isolated from infected cats.

## Introduction

Cats as most widespread animals contact directly with humans are reservoir hosts for some of intestinal parasitic infections. They have important role in contamination of environments, humans and animals by excreting eggs, cysts and oocysts of parasites in their stools (1–3).

A wide range of intestinal protozoa commonly infects cats throughout the world. There are some protozoa infections among feline intestinal parasites including *Toxoplasma gondii*, *Giardia duodenalis*, *Cryptosporidium* spp., *Sarcocystis* spp. and *Isospora felis* (*I. Felis*) and *rivolta (2).*

Among the coccidial parasites, *I. felis* and *Cryptosporidium* spp. infections are the most common (4). Almost all cats can be infected with *I. felis*. The pathogenicity of *I. felis* is, however, controversial (4).

Cystoisosporosis cause mild to severe diarrhea that may be bloody in puppies and kittens. The morbidity or mortality rates in severe cases are high (5).

*C. parvum* infection is a zoonosis that generally infects calves; however, it can also infect cats. *C. felis* has been reported in cats. Cryptosporidial infections are considered to be clinical importance in immunocompromised cats (4).

Microsporidia have been reported as causative agents of opportunistic infections especially in immunodeficient patients (6–8); however, are common in immunocompetent people (9). Diagnostic methods based on staining have been described in some previous studies (10, 11). Although numerous data associated with the epidemiology of Microsporidia infection exist which describe the zoonotic nature of the parasites, the information about transmission of infection from animals to humans needs more investigations (12).

Although a few studies about intestinal parasites in cat have been completed in different areas in Iran (13, 14), there has been no data on cat prevalence of protozoa infection in Meshkin Shahr.

This study aimed at identifying microsporidial and coccidian parasites isolated from domestic cats of Meshkin Shahr area, northwest of Iran using parasitological methods.

## Materials and Methods

In this cross-sectional study, 103 cats were caught by trapping (baited cage-traps) from different regions consist of Kojenagh, Ourkandi, Aghbelagh, Sarikhanlou of Meshkin Shahr, Ardabil province north-west of Iran during the Jun 2014 to Jun 2015. The information collected on each cat included age (78 young cats aged between 1–3 yr old and 25 cats aged >3 yr old) and gender (48 males and 55 females).

Two samples were collected from each cat, first sample was preserved in formalin (10%) and second one conserved in phosphate buffer saline (PBS) solution in Meshkin Shahr station of School of Public Health, Tehran University of Medical Sciences, and transferred to the Department of Medical Protozoology and Mycology, School of Public Health, Tehran University of Medical Sciences. The wet mount was prepared with PBS and formalin-ether concentration method was carried out for all the samples, and consequently observed under a light microscope with a final magnification of 400×.

### Acid Fast staining method

Formalin-ether concentration method was finalized, and smears were prepared from pellet of all samples and the slides allowed to dry at room temperature for 5 min following methanol fixation. Subsequently, all the samples were stained by a modified acid-fast staining method (15). Finally, all the slides were observed under the light microscope with a 1000× magnification.

### Preservation in dichromate potassium

Preservation in dichromate potassium (2.5%) was performed for all coccidian positive samples that collected in PBS solution and then sporulated coccidian oocysts were investigated

### Aniline Blue Staining Method

Sample smears were prepared, and after drying and methanol fixation, aniline blue staining method was carried out according to Ryan method (11). All the samples were observed with 1000× objective lens and evaluated for the detection of microspora spores.

### Ethical approval

This study was reviewed and approved by the Ethics Committee of Tehran University of Medical Sciences (Ethic no. 25287) in accordance with Helsinki Declaration and guidelines.

### Data analysis

Descriptive statistical methods relative to absolute and relative frequencies of microsporidial and coccidian intestinal parasites and their distribution in different areas of Meshkin Shahr district were done using SPSS (version 21) (Chicago, IL, USA).

## Results

**Fig. 1:**
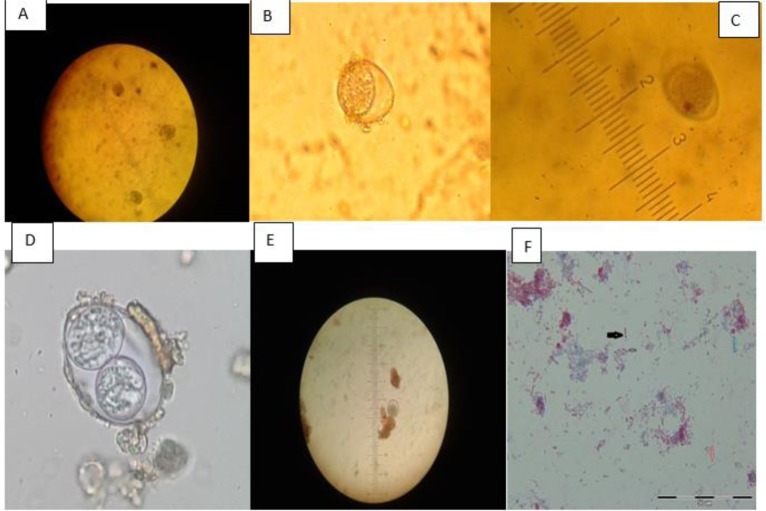
**A, B, C:** *Isospora* spp in concentrated samples, wet mount (400×); **D:** Sporulated *Isospora* spp oocyct contains two sporocysts after preservation in dichromate potassium; **E:** *Isospora* spp in wet mount (400×), **F:** Appearance of microsporidial spores (black arrow), yeast (blue arrow), bacterial element (red arrow) in aniline blue staining method (1000× magnification, original pictures)

One hundred and three stool samples from cats including 48 males and 55 females were collected. Generally, protozoa infections were attributed to *Isospora* spp. 6/103(5.8%), involving 4 female and 2 male cats. They were of different sizes and shapes. Large oocyst with a bulge on one side and a size of 38–51 by 27–39 μm that was similar to *I. felis* (Fig. 1A, B, C), and one medium oocyst that was more rounded and smaller than the previous ones and having a size of 18–28 μm by 16 – 23 (Fig. 1 E). Finally *Isospora* spp. has been identified because of lack of confirmation by molecular method. Three *Isospora* positive cases belonged to cats collected from Ourkandi and 3 cats collected from Kojenagh. The most positive cases of *Isospora* spp. (4/6) were seen in cats more than 3 yr old.

Result of *Isospora* preservation in dichromate potassium 2.5% has been shown in (Fig. 1D). The sporulated *Isospora* spp. oocyst contains two sporocysts and 4 sporozoites after preservation in dichromate potassium. The result of acid-fast staining method showed no *Cryptosporidium* spp. oocysts in this study.

In aniline blue staining method for detecting microspora, ovoid, transluminant spores with a belt-like strip in the middle were observed, with a size of spores ranging between 0.7–2.0 μm. Against the microspora spores characterization, bacteria portray blue color and fungi is displayed red, but with different sizes and shapes (Fig. 1F).

Microsporidian spores were identified in 46 (44.6%) among all samples stained by the aniline blue staining method. Parasites spores were found in 29 female cat fecal samples and 17 male ones. The most positive cases were prominent in Kojenagh [22] and then Ourkandi [15] followed by Aghbelagh [7] and Sarikhanlou [2] (Table 1).

**Table 1:** Distribution of Microsporidial infection among 103 domestic cats in different areas of Meshkin Shahr, northwestern Iran during 2014–2015

***Region of infection***	***Female***	***Male***	***Total (Number )***	***Total (percent)***
Kojenagh	12	10	22	21.4
Ourkandi	10	5	15	14.6
Aghbelagh	5	2	7	6.8
Sarikhanlou	0	2	2	1.9
Total	27	19	46	44.6

## Discussion

Animals, especially dogs and cats, play an important role in communities all over the world. Cats are the reservoir hosts of parasites including protozoa and helminthes because of their provision of nutritional and biological conditions (13).

Although parasitic infections have been reported in rodents from Meshkin Shahr areas (3, 16). There are limited investigations regarding gastrointestinal protozoa, especially coccidial and microsporidial infection in cat in this area. In this study, coccidia infections in 103 cats caught from Meshkin Shahr were found as follow: *Isospora* spp. 6/103(5.8%). Contrary to our study, high levels of infections have been reported in stray cats in Zanjan Province.

*I. rivolta* (80%), *I. felis* (70%) and *T. gondii* (42%) were reported in stray cats (13). The prevalence of intestinal parasites may be different with respect to geographical regions, climate and soil conditions of the areas, the type of cat population and seasons (2).

The prevalence of intestinal parasite depending on rural or urban areas may vary. Protozoan infections are more common in urban areas than rural area (2).

In this study, considering the situation of the regions, different pattern was recorded where *Isospora* infection was less common than previous study in Zanjan (13). Notably, the high levels protozoan infection rates have been reported in some previous studies (17, 18). In a fecal examination survey, among 217 cats obtained from Illinois, *I. felis* oocysts (23%), *I. rivolta* oocysts in (24%), and *Toxoplasma* or *Besnoitia* oocysts (1%) were identified (17).

In southern Germany, the prevalence of protozoa infections in 100 kittens kept on a farm were *I. felis* (67.1 %), *I. rivolta* (48.6%), *Toxoplasma/Hammondia* (17.1 %), *C. parvum* (4.3%) and *Giardia* (1.4%). The prevalences of protozoa infection in indoor kitten were *I. felis* (46.6%), *I. rivolta* (33.3%), *Giardia* (6.6%) and *C. parvum* (3.3%). Differences existed in relation to the cats keeping conditions on farms and indoors and highest protozoa rate was related to *I. felis* (67.1%), in the cats keeping conditions on farm (18).

Prevalence rates of *T. gondii* (18.2%), *I. felis* (15.1%) and *G. duodenalis* (17.0%) have been reported from Aboriginal communities in the west Kimberley region of Western Australia (19). A survey of pups for sale at pet stores in Atlanta showed that 34% were infected with *Giardia*, even though none had diarrhea or clinical illness (20).

Conversely, low levels of protozoa infection have been reported in some studies (2, 21, 22), where these findings are consistent with that of our study. In a study, overall fecal prevalence of coccidia (1.4%) and *Giardia* species (0.58%) were found in USA (22).

*I. rivolta* (8.9%), *I. felis* (5.3%), *T. gondii*/*Hammondia hammondi* (1.2%), *Sarcocystis* spp. (1%), *G. duodenalis* (0.7%) were reported from 414 household cats in different counties from Transylvania, Romania (2). The results of our study showed the presence of *Isospora* spp. 6/103 (5.8%), in 103 collected cats, which is consistent with the previous studies (2, 21).

The prevalence *Isospora* spp. recognized in this study is in agreement with the data obtained by other researchers in different countries. Thus, in cats exhibiting clinical signs, *Isospora* was reported in 12% of stool samples in Chile (23) and 3% in England (24). In cats without clinical signs, the prevalence of *Isospora* spp. infection has been reported as 6.3% in Spain (25), 5.6% in Australia (26) and between 0.2% and 9.7% in USA (27, 28).

Microspora spores were identified in 46/103 (44.6%) of all samples stained by the aniline blue staining method. There have been few studies concerning microsporidia in cats in Iran. *E. bieneusi* was detected using molecular method in 3/26 (11.53%) fecal samples collected from cats in a study in Tehran, Iran (29), and (3/40) 7.5% of the specimens obtained from cats in Iran (30).

Microsporidial spores were detected in 29.4% (10/34) of cat fecal samples by MT stain in Portugal (31). Our result was somewhat higher than that of Lobo et al, and this might be attributed to differences in geographic criteria of the two countries.

*E. bieneusi* was molecularly identified in 5% (3/60) of cats in Germany (32), in 17% (8/46) of cats in USA (33) and 14.3% (1/7) of cat fecal samples by PCR in Japan (34).

Taking into account the limitation of this study, PCR was unavailable and only parasitological method was used in the identification of the parasites.

## Conclusion

Microsporidia with a prevalence of 44.6% were more prevalent than coccidia such as *Isospora* (5.8%) due to zoonotic characteristics of microsporidia, hence this must be considered.

## References

[1] Alves JM Magalhães V Matos MA. [Toxoplasmic retinochoroiditis in patients with AIDS and neurotoxoplasmosis]. Arq Bras Oftalmol. 2010;73(2):150–4. [Article in Portuguese] 20549044 10.1590/s0004-27492010000200010

[2] Mircean V Titilincu A Vasile C. Prevalence of endoparasites in household cat (*Felis catus*) populations from Transylvania (Romania) and association with risk factors. Vet Parasitol. 2010;171(1):163–6. 20381250 10.1016/j.vetpar.2010.03.005

[3] Zarei Z Mohebali M Heidari Z Helminth Infections of *Meriones persicus* (Persian Jird), *Mus musculus* (House Mice) and *Cricetulus migratorius* (Grey Hamster): A Cross-Sectional Study in Meshkin-Shahr District, Northwest Iran. Iran J Parasitol. 2016;11(2): 213–20. 28096855 PMC5236098

[4] Dubey JP. Intestinal protozoa infections. Vet Clin North Am Small Anim Pract. 1993;23(1):37–55. 8421888 10.1016/s0195-5616(93)50003-7

[5] Lindsay DS Dubey J Blagburn BL. Biology of *Isospora* spp. from humans, nonhuman primates, and domestic animals. Clin Microbiol Rev. 1997;10(1):19–34. 8993857 10.1128/cmr.10.1.19 PMC172913

[6] Weber R Bryan RT. Microsporidial infections in immunodeficient and immunocompetent patients. Clin Infect Dis. 1994;19(3):517–21. 7811872 10.1093/clinids/19.3.517

[7] Mirjalali H Mohebali M Mirhendi H Emerging Intestinal Microsporidia Infection in HIV(+)/AIDS Patients in Iran: Microscopic and Molecular Detection. Iran J Parasitol. 2014;9(2): 149–54. 25848379 PMC4386033

[8] Agholi M Hatam GR Motazedian MH. HIV/AIDS-associated opportunistic protozoal diarrhea. AIDS Res Hum Retroviruses. 2013;29(1):35–41. 22873400 10.1089/aid.2012.0119 PMC3537293

[9] Lores B Arias C Lòpez-Miragaya I Torres J Fenoy S del Aguila C. Molecular diagnosis of intestinal microsporidiosis in pediatric patients from Vigo (NW, Spain). Res Rev Parasitol. 2001;61:43–9.

[10] Khanaliha K Mirjalali H Mohebali M Tarighi F Rezaeian M. Comparison of three staining methods for the detection of intestinal *Microspora* spp. Iran J Parasitol. 2014; 9(4):445–51. 25759724 PMC4345082

[11] Ryan NJ Sutherland G Coughlan K A new trichrome-blue stain for detection of microsporidial species in urine, stool, and nasopharyngeal specimens. J Clin Microbiol. 1993;31(12):3264–9. 7508457 10.1128/jcm.31.12.3264-3269.1993 PMC266395

[12] Deplazes P Mathis A Weber R. Epidemiology and zoonotic aspects of microsporidia of mammals and birds. Contrib Microbiol. 2000;6:236–60. 10943515 10.1159/000060363

[13] Esmaeilzadeh M Shamsfard M Kazemi A Khalafi S Altome S. Prevalence of protozoa and gastrointestinal helminthes in stray cats in Zanjan province, north-west of Iran. Iran J Parasitol. 2009;4(3):71–5.

[14] Khademvatan S Abdizadeh R Rahim F Hashemitabar M Ghasemi M Tavalla M. Stray cats gastrointestinal parasites and its association with public health in ahvaz city, South Western of iran. Jundishapur J Microbiol. 2014;7(8):e11079. 25485047 10.5812/jjm.11079 PMC4255209

[15] Henriksen SA Pohlenz JF. Staining of cryptosporidia by a modified Ziehl-Neelsen technique. Acta Vet Scand. 1981;22(3–4):594–6. 6178277 10.1186/BF03548684 PMC8300528

[16] Mohebali M Zarei Z Khanaliha K Natural Intestinal Protozoa in Rodents (Rodentia: Gerbillinae, Murinae, Cricetinae) in Northwestern Iran. Iran J Parasitol. 2017; 12(3): 382–8. 28979348 PMC5623918

[17] Guterbock W Levine N. Coccidia and intestinal nematodes of East Central Illinois cats. J Am Vet Med Assoc. 1977;170(12):1411–3. 873847

[18] Beelitz P Göbel E Gothe R. [Fauna and incidence of endoparasites in kittens and their mothers from different husbandry situations in south Germany]. Tierarztl Prax. 1992;20(3):297–300. 1496526

[19] Meloni BP Thompson R Hopkins RM Reynoldson JA Gracey M. The prevalence of *Giardia* and other intestinal parasites in children, dogs and cats from aboriginal communities in the Kimberley. Med J Aust. 1993;158(3):157–9. 8450779 10.5694/j.1326-5377.1993.tb121692.x

[20] Stehr-Green JK Murray G Schantz PM Wahlquist SP. Intestinal parasites in pet store puppies in Atlanta. Am J Public Health. 1987;77(3):345–6. 3812843 10.2105/ajph.77.3.345 PMC1646912

[21] Blagburn BL Lindsay DS Vaughan JL Prevalence of canine parasites based on fecal flotation. The Compendium on continuing education for the practicing veterinarian (USA). 1996 http://www.nal.usda.gov/

[22] De Santis-Kerr AC Raghavan M Glickman NW Prevalence and risk factors for *Giardia* and coccidia species of pet cats in 2003–2004. J Feline Med Surg. 2006;8(5):292–301. 16678461 10.1016/j.jfms.2006.02.005 PMC10822243

[23] Lopez J Abarca K Paredes P Inzunza E. [Intestinal parasites in dogs and cats with gastrointestinal symptoms in Santiago, Chile]. Rev Med Chil. 2006;134(2):193–200. 16554927 10.4067/s0034-98872006000200009

[24] Tzannes S Batchelor DJ Graham PA Pinchbeck GL Wastling J German AJ. Prevalence of *Cryptosporidium*, *Giardia* and *Isospora* species infections in pet cats with clinical signs of gastrointestinal disease. J Feline Med Surg. 2008;10(1):1–8. 17706446 10.1016/j.jfms.2007.05.006 PMC10911146

[25] Miró G Montoya A Jiménez S Frisuelos C Mateo M Fuentes I. Prevalence of antibodies to *Toxoplasma gondii* and intestinal parasites in stray, farm and household cats in Spain. Vet Parasitol. 2004;126(3):249–55. 15567588 10.1016/j.vetpar.2004.08.015

[26] Palmer CS Thompson RA Traub RJ Rees R Robertson ID. National study of the gastrointestinal parasites of dogs and cats in Australia. Vet Parasitol. 2008;151(2):181–90. 18055119 10.1016/j.vetpar.2007.10.015

[27] Carleton RE Tolbert MK. Prevalence of *Dirofilaria immitis* and gastrointestinal helminths in cats euthanized at animal control agencies in northwest Georgia. Vet Parasitol. 2004;119(4):319–26. 15154596 10.1016/j.vetpar.2003.10.019

[28] Shukla R Giraldo P Kraliz A Finnigan M Sanchez AL. *Cryptosporidium* spp. and other zoonotic enteric parasites in a sample of domestic dogs and cats in the Niagara region of Ontario. Can Vet J. 2006;47(12):1179–84. 17217087 PMC1636587

[29] Askari Z Mirjalali H Mohebali M Molecular Detection and Identification of Zoonotic Microsporidia Spore in Fecal Samples of Some Animals with Close-Contact to Human. Iran J Parasitol. 2015;10(3):381–8. 26622293 PMC4662738

[30] Jamshidi S Tabrizi AS Bahrami M Momtaz H. Microsporidia in household dogs and cats in Iran; a zoonotic concern. Vet Parasitol. 2012;185(2):121–3. 22035849 10.1016/j.vetpar.2011.10.002

[31] Lobo ML Teles A Barao DA Cunha M Microsporidia detection in stools from pets and animals from the zoo in Portugal: a preliminary study. J Eukaryot Microbiol. 2003;50(6):581–2. 14736171 10.1111/j.1550-7408.2003.tb00638.x

[32] Dengjel B Zahler M Hermanns W Zoonotic potential of *Enterocytozoon bieneusi*. J Clin Microbiol. 2001;39(12):4495–9. 11724868 10.1128/JCM.39.12.4495-4499.2001 PMC88572

[33] Santín M Trout JM Vecino JAC Dubey J Fayer R. *Cryptosporidium*, *Giardia* and *Enterocytozoon bieneusi* in cats from Bogota (Colombia) and genotyping of isolates. Vet Parasitol. 2006;141(3):334–9. 16860480 10.1016/j.vetpar.2006.06.004

[34] Abe N Kimata I Iseki M. Molecular evidence of *Enterocytozoon bieneusi* in Japan. J Vet Med Sci. 2009;71(2):217–9. 19262036 10.1292/jvms.71.217

